# Lumbar disc herniation reabsorption: a review of clinical manifestations, mechanisms, and conservative treatments

**DOI:** 10.3389/fmed.2025.1633762

**Published:** 2025-07-28

**Authors:** Yuan Chai, Xuyu Shen, Zixin Wang, Xiaoyun Zhang, Zhiqiang Wang, Xinchen Zuo, Jintao Liu

**Affiliations:** ^1^Suzhou TCM Hospital Affiliated to Nanjing University of Chinese Medicine, Suzhou, Jiangsu, China; ^2^Nanjing University of Chinese Medicine, Nanjing, Jiangsu, China; ^3^Ruikang Hospital Affiliated with Guangxi University of Chinese Medicine, Nanning, Guangxi, China; ^4^Guangxi University of Chinese Medicine, Nanning, Guangxi, China; ^5^Suzhou Academy of Wumen Chinese Medicine, Suzhou, Jiangsu, China

**Keywords:** lumbar disc herniation, reabsorption, mechanisms of action, treatment, research progress

## Abstract

Lumbar disc herniation (LDH) is a common and frequently occurring condition primarily caused by lumbar intervertebral disc degeneration (LIVDD) and protrusion of the nucleus pulposus (NP), with low back pain and lower limb pain as the main clinical manifestations. It is characterized by a prolonged disease course and a high recurrence rate, with patients often experiencing long-term suffering, significantly impairing their quality of life and mental health. Studies have found that some LDH patients, without undergoing surgery or chemonucleolysis, experience spontaneous shrinkage and resorption of the intervertebral disc (IVD) tissue, along with relief of back and leg pain-a phenomenon referred to as LDH reabsorption. Modern medical research suggests that this reabsorption process is closely related to factors such as inflammatory responses, macrophage activation, extracellular matrix enzyme balance, neovascularization, ferroptosis, mitochondrial function, oxidative stress, and cellular autophagy. However, the precise mechanisms of LDH reabsorption and commonly used clinical therapies remain unclear, leading to suboptimal treatment outcomes. This study systematically reviews the relevant literature on LDH reabsorption, focusing on clinical diagnosis, underlying mechanisms, and common therapeutic strategies, aiming to summarize recent research progress and provide theoretical references for future clinical prevention and treatment of LDH.

## 1 Introduction

Lumbar disc herniation (LDH) is a syndrome primarily characterized by low back pain (LBP) and lower extremity pain, resulting from nerve root and cauda equina compression and irritation due to lumbar intervertebral disc degeneration (LIVDD), rupture of the annulus fibrosus (AF), and protrusion of the nucleus pulposus (NP) (1). Due to its chronic course, high recurrence rate, and poor prognosis-with severe cases even resulting in lower limb paralysis and urinary incontinence (2)-LDH imposes a heavy economic burden on families and society. Currently, authoritative epidemiological studies on LDH are lacking; however, numerous studies have demonstrated that the prevalence of LDH increases with age and is closely related to the widespread use of computers and changes in work patterns (3). Recent studies have revealed a phenomenon termed “LDH reabsorption,” in which patients experience spontaneous shrinkage and absorption of intervertebral disc (IVD) tissue, along with alleviation of low back and leg pain, despite receiving no surgical intervention or chemonucleolysis (4). Several clinical studies have gradually confirmed that the phenomenon of LDH reabsorption is widely present in patients with LDH (5, 6), and is associated with factors such as the patient’s age, sex, and body weight. Younger patients are more likely to experience reabsorption compared to older patients. Due to differences in hormone levels, females, influenced by estrogen, have a lower probability of reabsorption than males. Patients with lower body weight are more likely to undergo reabsorption than those with higher body weight (7, 8). Additionally, the larger the protrusion of the IVD tissue, the higher the likelihood of reabsorption occurring. In summary, LDH reabsorption occurs across all age groups in both male and female patients and is present throughout the entire process of the onset and progression of LDH disease. Therefore, studying the mechanisms of LDH reabsorption and its clinical diagnosis and treatment holds significant value.

In systematically reviewing recent studies related to LDH reabsorption, the author found that its occurrence is closely associated with the size of the disc protrusion in LDH, which can be confirmed by imaging techniques such as computed tomography (CT) and magnetic resonance imaging (MRI). Its underlying mechanisms are closely related to inflammatory reactions, tissue vascularization, tissue dehydration, hematoma absorption, autoimmune responses, and cellular autophagy. Therapies such as bed rest, pharmacological treatment, exercise therapy, and epidural injections can facilitate this reabsorption process (9, 10). This article systematically analyzes LDH reabsorption from perspectives of clinical diagnosis, underlying mechanisms, and therapeutic approaches, aiming to provide insights and references for exploring the significance of LDH reabsorption in the clinical management of LDH.

## 2 Search method

**Figure 1:**
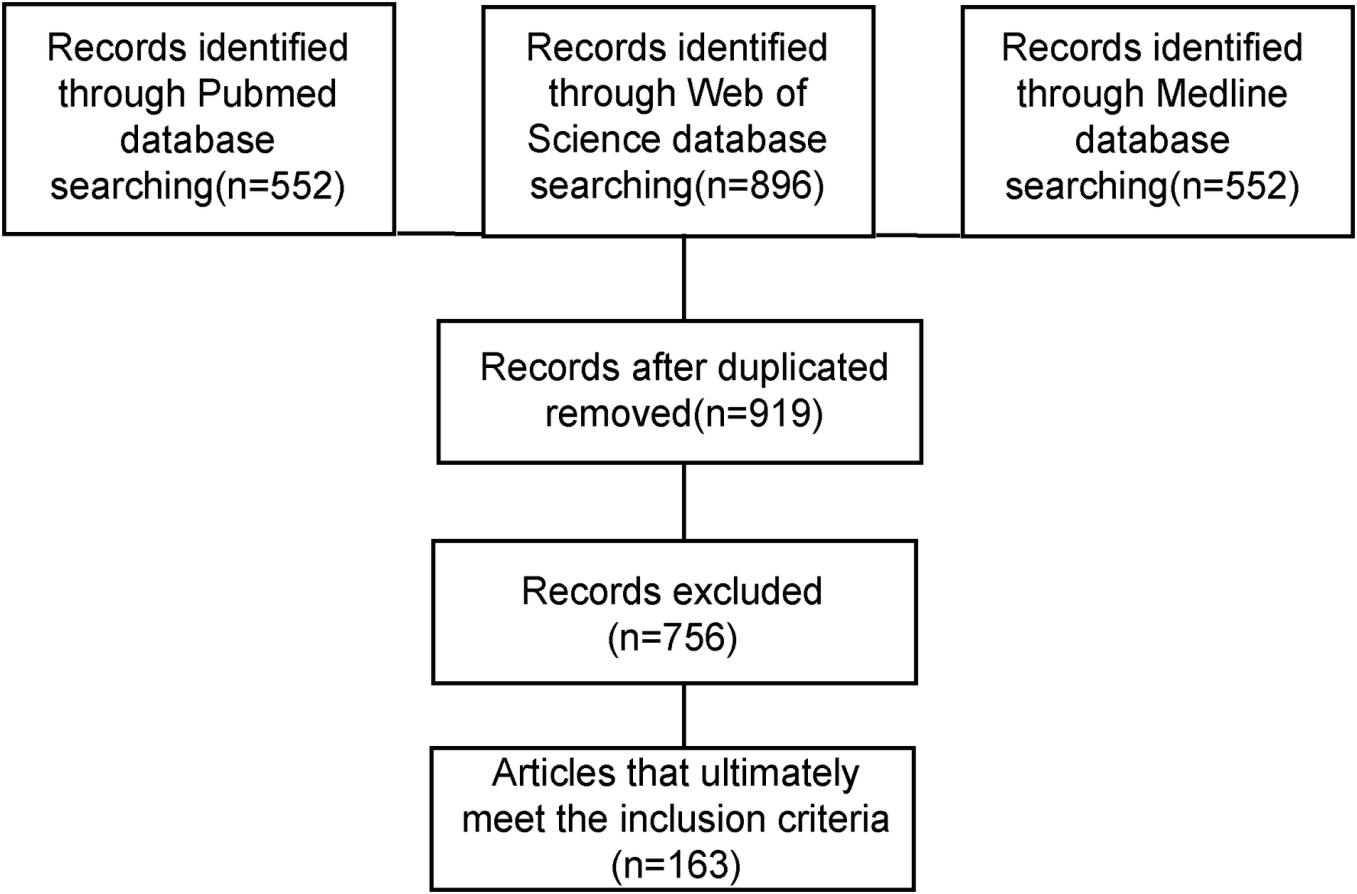
Flowchart of literature inclusion process.

We searched for all reports published in English in the PubMed database from January 1980 to April 2025. The literature search was conducted from May 1 to May 10, 2025. The following medical subject headings were used: (“lumbar disc herniation” OR “lumbar disc protrusion”) AND (“disease classification” OR “imaging findings” OR “common onset time” OR “mechanisms” OR “conservative treatment” OR “spontaneous regression” OR “resorption” OR “natural resolution” OR “herniation regression”). Additionally, we reviewed the references from the extracted papers, and after de-duplication, two independent reviewers screened the titles and abstracts. Studies whose titles and abstracts did not meet the inclusion criteria were excluded from this review. After careful screening, a total of 163 articles were finally included in the review, as shown in Figure 1.

### 2.1 Clinical diagnosis of LDH reabsorption

#### 2.1.1 Common classification of LDH reabsorption

The occurrence of LDH reabsorption is closely related to the structure of IVD tissue. The protruded IVD tissue includes the AF, cartilage endplate (CEP), and NP. The reabsorption phenomenon is closely related to the water content of the NP, whereby gradual dehydration leads to a reduction in the volume of the protruded IVD tissue (11). The likelihood of LDH reabsorption is closely associated with clinical classification. Relevant studies have combined clinical experience with experimental analyses to evaluate the relationship between LDH reabsorption and LDH classification. MRI is utilized to detect bone marrow lesions in vertebrae adjacent to the CEP. Factors indicating reabsorption are identified through darker signals on T1-weighted (T1W) imaging and brighter contrasts on T2-weighted (T2W) imaging. LDH is classified into non-ruptured types, including degenerative, bulging, protruded, and subligamentous types, and ruptured types. Normal IVD tissue is avascular, which separates the NP from the host immune system and suppresses the infiltration of immune cells and cytokines. In non-herniated IVD tissue, the morphology remains intact. However, when herniation occurs, the blood and NP barrier is damaged, exposing the IVD to the immune microenvironment, triggering an autoimmune response, and leading to various pathological processes, such as angiogenesis and immune cell infiltration (12). These mechanisms all play a role in the reabsorption of non-herniated LDH. The posterior longitudinal ligament type and free-type 2 are classified as ruptured types (13, 14). Compared to non-ruptured types, ruptured types are more likely to undergo reabsorption, with a higher incidence in free-type patients than protruding-type patients, and a higher incidence in lateral-type compared to central-type (11). Under the same conservative treatment, the reduction rate in free-type LDH patients is higher than that in the ligamentous and subligamentous types (15). The likelihood of absorption is higher in large protrusions compared to medium and small protrusions, and patients with normal intervertebral spaces are more likely to experience reabsorption than those with narrowed intervertebral spaces (16). Extruded LDH has a larger contact surface area for macrophage adhesion, resulting in a higher number of macrophages compared to protruding LDH (17). Moreover, in ruptured-type LDH, the NP begins to contact external tissues, making it more likely to trigger the body’s immune-inflammatory response and angiogenesis. This, in turn, induces inflammatory responses and vascularization mechanisms. Additionally, cell membrane rupture and necrosis lead to the occurrence of ferroptosis, which, together with various mechanisms, promotes LDH reabsorption (18–20).

In summary, LDH resorption occurs at various stages of LDH, but is most common in ruptured-type LDH. The larger the area of Intervertebral Disc Herniation (IDH), the faster the resorption rate. In the early stages of LDH, ligamentous-type and free-type protrusions cause the annulus fibrosus to rupture and form hematomas in the epidural venous plexus. MRI shows that the reduction in protruding IVD is associated with hematoma resorption and dehydration of the NP after it protrudes into the epidural space. LDH reabsorption or reduction is more common in free-type, posterior longitudinal ligament-type, and ruptured-type LDH. This is related to whether the posterior longitudinal ligament is intact, the amount of NP in the epidural space, and the distance of the free NP. The larger the protruding NP, the more NP components protrude into the epidural space, and the further the free NP, the more likely resorption occurs (11, 21). Therefore, imaging examination is of significant importance in assessing LDH resorption.

#### 2.1.2 Imaging manifestations of LDH reabsorption

**Figure 2:**
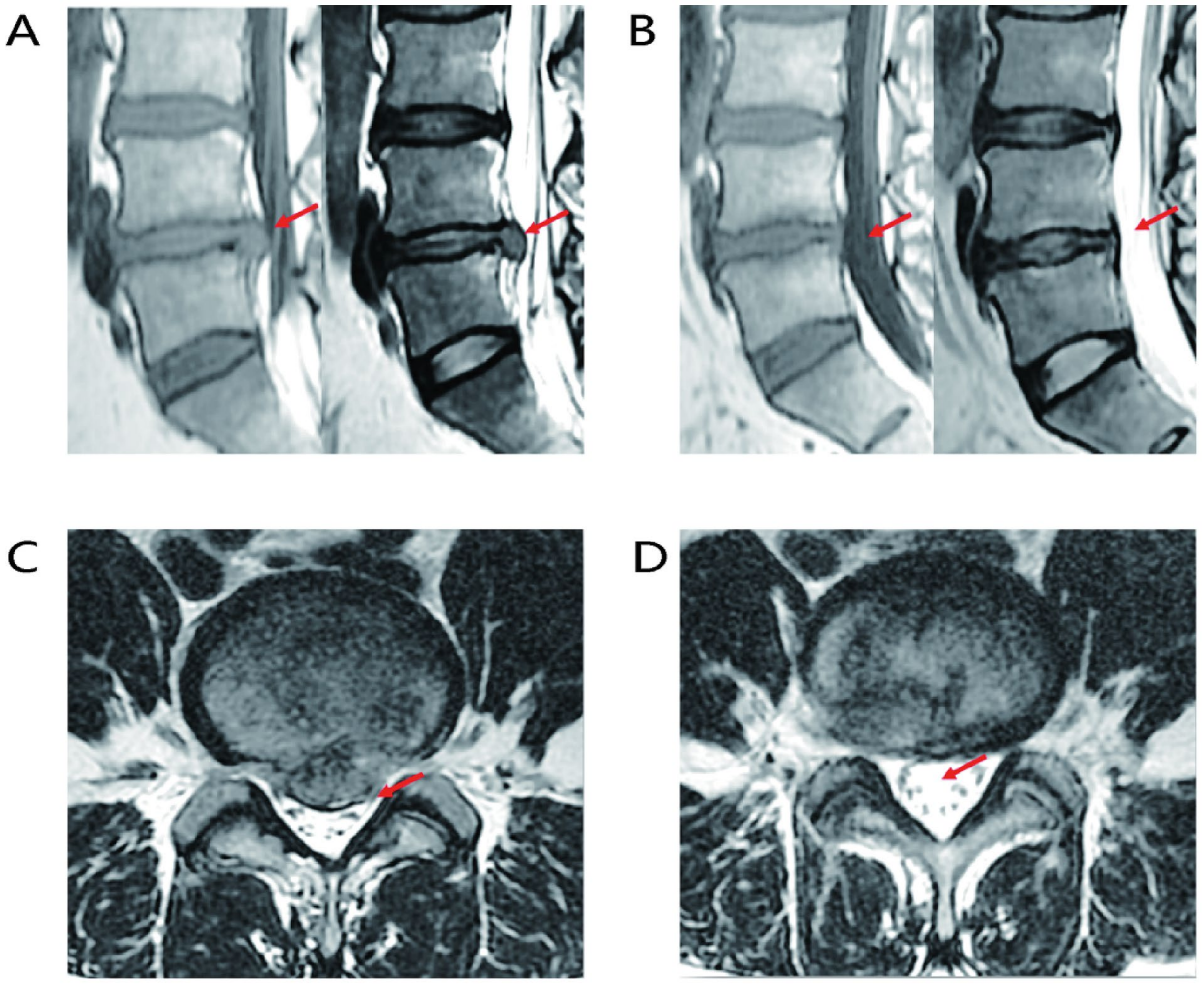
Representative MRI images of LIVD reabsorption. **(A)** Sagittal MRI image of LIVD before treatment. **(B)** Sagittal MRI image after LIVD reabsorption. **(C)** Axial MRI image of LIVD before treatment. **(D)** Axial MRI image after LIVD reabsorption.

The proposal of the LDH reabsorption theory is closely associated with advancements in imaging technologies. LDH reabsorption was first observed through CT in 1984 and subsequently received more extensive investigation with the advancement of MRI techniques (22–24), gradually becoming an essential standard for diagnosing LDH and related lumbar intervertebral disc (LIVD) (25). MRI images demonstrate that as the peripheral signal ring around the protrusion intensifies, greater edge thickness corresponds to higher signal intensity. Significant enhancement of reabsorption at the margins of protruded tissue is an important factor for evaluating spontaneous regression of IDH (26–28), as shown in Figure 2. LDH tissues have a high cartilage content, and subtle changes associated with reabsorption can be detected by MRI. During the natural progression of LDH, imaging features of protruded tissues play a critical role in determining reabsorption status. When LDH tissue has a relatively high cartilage component or when MRI displays only subtle changes, spontaneous LDH absorption is usually impeded. Conversely, if contrast-enhanced MRI reveals free IVD fragments breaking through into the epidural space, an autoimmune response can be stimulated, inducing inflammation and subsequently leading to peripheral granulation tissue formation with annular enhancement. Notably, the non-enhancing free IVD fragment, known as the “bull’s eye sign,” serves as an important imaging marker predictive of favorable LDH reabsorption. Additionally, the greater the proportion of protruded IVD tissue within the spinal canal, the higher the likelihood of reabsorption (29). In summary, MRI is crucial for the diagnosis and evaluation of LDH reabsorption.

#### 2.1.3 Favorable time for LDH reabsorption

Clinical observations have shown that over time, all patients with LDH exhibited resorption of the protruded IVD tissue on CT imaging, accompanied by clinical symptom improvement, and in some cases, complete resolution (30). Follow-up CT scans revealed that in conservatively treated LDH patients, the protruded IVD retracted to some extent, with a reduction exceeding 50% in most cases (31). This phenomenon occurred more frequently within the first 3 months (32). After 6 months of conservative treatment, the NP protrusion in most patients either completely resolved or decreased in size, with corresponding improvement in clinical symptoms (33). With the widespread clinical application of MRI, research on LDH resorption from both clinical and imaging perspectives has become increasingly precise and comprehensive. MRI observations have confirmed that the early stage of LDH represents a period of active resorption, with the degree of resorption correlating with improvements in clinical symptoms and signs (21). As clinical diagnosis and treatment have improved, the understanding of LDH resorption has become more refined beyond initial CT-based studies.

In related clinical studies (15, 21, 34–39) MRI was used for regular follow-up of LDH patients, measuring parameters such as the volume of the herniated disc, the extent of marginal thickening, and the percentage of thickening relative to the original disc height for a comprehensive assessment of treatment efficacy. The follow-up periods in these studies have been progressively shortened from 8 to 77 months (as in earlier CT studies) to 1–28 months. Ultimately, most clinical studies indicate that LDH resorption commonly begins after 2 months, typically occurring within the first 2–12 months, with the 3–6 month period being the most frequent, as shown in Table 1.

**Table 1:** LDH reabsorption incidence and onset time imaging studies.

Follow-up method	Percentage of people with LDH resorption	Onset time	References
CT follow-up	100%	5–36 months	Teplick and Haskin (30)
	91.67%	8–77 months	Saal et al. (31)
	100%	0–3 months	Fagerlund et al. (32)
	67%	≥6 months	Delauche-Cavallier et al. (33)
MRI follow-up	100%	≈5 months	Komori et al. (21)
	69.44%	1–28 months	Ahn et al. (15)
	39.06%	6 months	Splendiani et al. (35)
	93.33%	5–56 months	Cribb et al. (36)
	8.62%	0–12 months	Martinez-Quinones et al. (37)
	100%	0–2 months	Autio et al. (38)
	100%	2–12 months	Autio et al. (39)

### 2.2 Mechanism of action of LDH reabsorption

#### 2.2.1 Mechanisms of inflammatory response in LDH reabsorption

The IVD is an avascular, enclosed structure composed of three parts: the AF, the cartilage endplate (CEP), and the NP. The AF is a ligament-like lamellar structure composed of type I collagen fibers that encircle the NP. The CEP consists of a small amount of hyaline cartilage located between the vertebral endplate and the NP. A unique blood–nucleus pulposus barrier, along with the expression of Fas ligand (FasL), isolates the NP from the immune system. When IVD degeneration occurs or is influenced by an inflammatory microenvironment, it often leads to discogenic pain (40). IVD disorders are not caused by a single factor, and some patients with LBP caused by intervertebral disc degeneration (IVDD) continue to experience chronic pain even after lumbar IVD discectomy. This persistent pain is closely related to inflammation-induced remodeling of the endplate, which is rich in sensory nerve endings (41).

Machine learning analysis indicates that LDH is influenced by inflammatory cell infiltration and the release of atypical cytokines. When copper ion levels gradually exceed a certain threshold, the resulting cellular hyper-respiration increases cytotoxicity, ultimately leading to copper-induced cell death. The elevated immune infiltration observed in LDH patients is closely associated with copper-related gene (CRG) clusters that regulate this process (42). Both LDH and LBP-related disorders are strongly linked to inflammation and may result in adverse symptoms such as stimulation of nociceptive nerve fibers (19). In clinical practice, patients with protruding or free-type LIVD exhibit significantly elevated levels of inflammatory mediators such as Tumor Necrosis Factor-*α* (TNF-α), Interleukin-1 beta (IL-1β), and Interleukin-6 (IL-6) (43, 44). Pro-inflammatory factors such as TNF-α stimulate the IVD to produce chemokines, inducing the activation of Matrix Metalloproteinases (MMPs), which indirectly promotes angiogenesis, leading to a functional inflammatory response that facilitates LDH reabsorption. The latter secretes anti-inflammatory factors such as Interleukin-4 and Interleukin-10, which promote phagocytosis and reduce the inflammatory response, thereby promoting IVD reabsorption (45). Therefore, the inflammatory response can further promote LDH reabsorption by activating MMPs and promoting angiogenesis. Furthermore, postoperative levels of IL-1β and IL-6 are significantly elevated in the herniated regions and spinal nerves in both the puncture and lateral puncture groups (46). When herniated IVD tissue breaches the AF, it is recognized as an antigen by the immune system, triggering an autoimmune response. Research on demethoxycurcumin has revealed its therapeutic potential for inflammation-related diseases through immunomodulation. It achieves this by suppressing pro-inflammatory cytokines via inhibition of the mitogen-activated protein kinase (MAPK)/nuclear factor-κB (NF-κB) signaling pathway and related transcriptional activators (47). Additionally, TNF-*α* and IL-1β secreted by macrophages activate the p38MAPK signaling pathway (48, 49), which further promotes inflammatory mediator expression and contributes to the development of radicular pain (50). When NP tissue protrudes into the epidural space, it triggers an autoimmune response, leading to a significant increase in pro-inflammatory factors such as TNF-*α*, IL-1β, and IL-6. This, in turn, results in immune cell infiltration. The recruited immune cells interact with the intervertebral disc cells to secrete various factors that promote LDH reabsorption.

#### 2.2.2 Mechanisms of macrophage triggering in LDH reabsorption

Macrophages, as key immune regulatory cells, play an important role in the infiltration and activation of LDH reabsorption. Both protruded and free LDH exhibit abundant macrophage infiltration, which is closely related to the differentiation of macrophage precursors into classically activated M1-type and alternatively activated M2-type macrophages. M1-and M2-type cytokines undergo phenotypic and functional differentiation under the influence of various cytokines. M1-type macrophages primarily produce pro-inflammatory cytokines such as TNF-*α*, IL-6, and IL-1β, which regulate the inflammatory response that mediates pain, and are closely associated with IVDD and sciatica. M2-type macrophages possess anti-inflammatory and healing-promoting functions, playing roles in tissue repair, fibrosis, and tissue regeneration by modulating functional inflammatory effects. Studies have shown that anti-inflammatory cytokines secreted by M2-type macrophages, such as IL-4 and IL-10, stimulate protruded IVD absorption by promoting phagocytosis and reducing inflammation (51, 52). Cytokines and chemokines secreted by IVD cells and macrophages in the IDH, such as Monocyte Chemoattractant Protein (MCP)-1, MCP-3, MCP-4, Chemokine (C-C motif) ligand 5, Macrophage Inflammatory Protein-1*α* (MIP-1α), and Interferon-*γ*-inducible protein 10, promote the chemotaxis of macrophages. When LDH occurs, IVD cells rapidly produce TNF-α, IL-1β, and MCP-1 (53), thereby recruiting macrophages. The produced TNF-α works in conjunction with MMP-3 produced by chondrocytes to promote the release of chemotactic factors and macrophage migration, further enhancing IVD cell reabsorption (54). Additionally, the protruded IVD is unable to interact with complexes formed by Transforming Growth Factor-beta (TGF-*β*) and Extracellular Matrix (ECM) foundation molecules, preventing the inhibition of the NF-κB signaling pathway. This, in turn, upregulates the level of Thymic Stromal Lymphopoietin (TSLP) in IVD tissue, which promotes MCP-1 expression through the regulation of the PI3K/Akt signaling pathway. When stimuli promote the recruitment of macrophages to the IVD, the protruded IVD interacts with macrophages to induce the production of inflammatory cytokines (55). This initiates immune-inflammatory responses and phagocytosis, along with the alternating facilitation of reabsorption by M1 and M2 macrophages, ultimately inducing LDH reabsorption.

#### 2.2.3 Mechanism of matrix enzyme metabolic balance in LDH reabsorption

The development of IVDD is closely associated with mitochondrial dysfunction and ECM metabolic imbalance in nucleus pulposus cells (NPCs), caused by the excessive accumulation of reactive oxygen species (ROS). MMPs, a group of zinc-dependent intracellular proteases, are involved in the degradation of ECM and other proteins and have potential roles in tissue remodeling. The expression of MMP-1 in the IVD is significantly associated with histological degeneration in patients with cervical spondylosis and IVDD. MMP-1 is the most abundant and predominant catabolic enzyme involved in IVD degeneration (56), playing a key role in the pathogenesis of IVDD. As a group of dependent enzymes, MMPs facilitate tissue resorption and ECM remodeling. In protruded IVD tissue, infiltrating macrophages secrete pro-inflammatory cytokines such as IL-1 and TNF-*α*, which in turn upregulate MMP expression and accelerate proteoglycan loss in the protruded disc. Among the MMP family, upregulation of MMP-3 promotes the release of macrophage chemotactic factors, thereby enhancing macrophage infiltration, proteoglycan degradation, and IVD resorption (54). Studies have shown that in patients with intervertebral disc herniation (IDH), the activity of matrix-degrading enzymes in the IVD increases while the activity of inhibitory enzymes decreases, thereby accelerating the resorption of protruded IVD tissue (57). MMPs contribute to resorption by regulating macrophage infiltration, modulating inflammatory mediators, and maintaining tissue and cellular homeostasis. Recombinant human MMP-7 (rhMMP-7) has already been applied in the treatment of IVD degeneration, offering a minimally invasive alternative that partially avoids the complications associated with surgical intervention (58, 59). On one hand, the upregulation of MMP expression induced by TNF-*α* accelerates the loss of proteoglycans in the protruded IVD and promotes the degradation of the cartilage matrix. On the other hand, the secretion of MMPs is involved in the complex interaction between macrophages and chondrocytes in LDH reabsorption (60). The contact between macrophages and the IVD promotes a cascade reaction between macrophages and chondrocytes. MMP-3 and MMP-7 play a role in the macrophage-chondrocyte interaction, indirectly affecting cartilage matrix degradation and disc absorption.

#### 2.2.4 Mechanism of vascularization factors in LDH reabsorption

IVD tissue lacks a direct blood supply, and its nutrients are primarily derived from diffusion through the CEP and AF. The degree of vascularization in herniated NP tissue is closely correlated with the extent of herniation resorption. When ruptured IVD material enters the epidural space, neovascularization facilitates macrophage infiltration and phagocytosis, resulting in the reduction or even disappearance of the herniated tissue (61, 62), thereby promoting LDH reabsorption (63). Clinical studies have shown that during the progression of IVDD, the polarization state of macrophages surrounding neovessels may change, with an increased presence of CD16 + perivascular macrophages being associated with chronic inflammation (64). Pro-angiogenic mediators secreted by different macrophage subtypes are key regulators of neovascularization during inflammation. TNF-*α* secreted by macrophages activates the NF-κB signaling pathway, upregulating the expression of vascular endothelial growth factor (VEGF) and thereby promoting neovessel formation (65). This angiogenic process involves early pro-inflammatory cytokines released by M1 macrophages, while anti-inflammatory M2 macrophages, which are associated with tissue regeneration, promote endothelial cell proliferation and stabilize vascular growth through the secretion of MMP-9 and platelet-derived growth factor-BB (PDGF-BB). Failure of M1-to-M2 repolarization results in persistent chronic inflammation, increased osteoclast activation, reduced osteoblast formation, and impaired stem cell function, ultimately diminishing bone regeneration capacity by increasing bone resorption and decreasing bone formation during healing (66–68). It can be concluded that macrophage infiltration in the IVD is involved in the regulation of angiogenesis. The formation of new blood vessels promotes macrophage infiltration and the regulation of M1 macrophages with pro-inflammatory effects and M2 macrophages with anti-inflammatory effects, thereby promoting endothelial cell growth and stabilizing blood vessel formation. This leads to the reduction or disappearance of the protruded IVD tissue, facilitating LDH reabsorption.

#### 2.2.5 The role of ferroptosis in the mechanism of LDH reabsorption

Ferroptosis is a newly identified form of iron-dependent cell death, distinguished from classical cell death modalities by its unique mitochondrial abnormalities-such as mitochondrial shrinkage, reduced cristae, and increased membrane density. It is driven by intracellular iron overload and accumulation of ROS-dependent lipid peroxides, leading to plasma membrane rupture, cytoplasmic content leakage, and eventual necrosis (20). Ferroptosis is closely associated with the NP tissue in LIVDD. *In vitro* studies have shown that homocysteine-induced oxidative stress can activate ferroptosis and cause substantial loss of NPCs (69). Moreover, NPCs apoptosis is strongly linked to the activation of the p38 MAPK signaling pathway, which promotes the reabsorption of ruptured LDH (70, 71). IL-6 in LIVDD can also induce oxidative stress in chondrocytes, disrupt iron homeostasis, and trigger ferroptosis, further contributing to NPCs-associated degeneration (72).

Experimental findings further support that tert-butyl hydroperoxide can induce ferroptosis in AF cells and NPCs, characterized by downregulation of glutathione peroxidase 4 (GPX4) and ferritin heavy chain, and upregulation of cyclooxygenase-2 (COX-2) and acyl-CoA synthetase long-chain family member 4 (ACSL4) (73). Wang et al. (74) observed that in mice fed with high-iron diets, CEP cells exhibited decreased GPX4 and SLC7A11 expression, increased ROS production, elevated 4-hydroxynonenal levels, and enhanced lipid peroxidation, suggesting iron overload-mediated oxidative stress as a key driver of ferroptosis in CEP cells. Additionally, in LDH patients, ruptured peripheral vasculature surrounding NP tissues results in erythrocyte extravasation and elevated heme levels. Heme breakdown further increases local ROS and reduces GPX4, promoting ferroptosis in NPCs and thereby accelerating LIVDD progression (75). The newly formed NP blood vessels may expose tissues to a large amount of hemoglobin, thereby triggering cytotoxicity and ferroptosis, accelerating progressive degeneration. When iron ions excessively accumulate in cells and tissues, they disrupt the redox balance, catalyzing ROS production and promoting ferroptosis. Related studies have shown that when ferritin autophagy or BMSCs ferroptosis is inhibited, cell viability increases, BMSCs dysfunction is reduced compared to before, and bone damage is reversed (76).

#### 2.2.6 Mechanism of mitochondrial action in LDH reabsorption

Mitochondria are highly dynamic membrane-bound organelles that provide the majority of the chemical energy required for biochemical reactions in eukaryotic cells. Cells maintain mitochondrial health through a mitochondrial quality control mechanism, which includes selectively removing damaged mitochondria and balancing mitochondrial biosynthesis. These organelles communicate with the cell nucleus and other cellular structures to help maintain cellular homeostasis, enabling the cell to adapt to stress, promote development, and support metabolism, energy, and genetic regulation within the cell (77). Mitochondrial fission and fusion not only safeguard mitochondrial genetics and function but are also intimately linked to energy metabolism, aging, and cell death (78, 79). Accordingly, mitochondria act as central regulators of apoptosis, autophagy, and necroptosis. Excessive mitochondrial fission activates the intrinsic apoptotic pathway, up-regulating the pro-apoptotic proteins caspase-3, caspase-9, and Bax while down-regulating the anti-apoptotic protein Bcl-2, thereby triggering apoptosis of NPCs (80). Moreover, mitochondrial DNA (mtDNA), acting as a damage-associated molecular pattern, activates the TLR9–NF-κB–NLRP3 signaling cascade, promotes NLRP3 inflammasome expression, and induces pyroptosis of NPCs, thus facilitating the reabsorption of LDH (81). Given that NPCs apoptosis is pivotal in IVDD-with compelling evidence highlighting the mitochondrial pathway as a key mediator (82, 83). In summary, NPC apoptosis is closely associated with LDH reabsorption, and mitochondrial activation and apoptosis play critical roles in the initiation and progression of IVDD. When mitochondrial dysfunction leads to cellular impairment and reduced activity, mitochondrial regulatory function becomes unbalanced in degenerated NPCs. Excessive activation of degenerative cells leads to the mitochondrial unfolded protein response, initiating non-selective mitochondrial autophagy to salvage damaged mitochondria. Over-activation exacerbates mitochondrial dysfunction. Mitochondria in NPCs are more vulnerable to cellular interference, resulting in mitochondrial dysfunction (84). When the mitochondrial respiratory chain is disrupted, excessive ROS triggers oxidative stress, disrupting the proton gradient and the stability of the adenosine nucleotide phosphorylation process. Antioxidants may enhance mitochondrial antioxidant capacity by neutralizing free radicals and alleviating oxidative stress, thus reducing oxidative stress on mitochondria, improving the survival and regeneration of IVD cells, and promoting LDH reabsorption.

#### 2.2.7 Mechanism of oxidative stress in LDH reabsorption

Oxidative stress refers to the accumulation of ROS resulting from an imbalance between oxidants and antioxidants within cells and tissues. This redox disequilibrium-triggered by excessive production of oxygen free radicals, reactive metabolites, or oxidants relative to antioxidant defense mechanisms-damages essential biomolecules and cellular structures, posing potential harm to the organism. It has been closely implicated in the pathogenesis of numerous diseases, including IVDD and atherosclerosis (85, 86). In the context of disrupted IVD homeostasis, oxidative stress primarily exerts its deleterious effects by inhibiting the synthesis of ECM by NPCs, promoting the secretion of matrix-degrading enzymes and proinflammatory cytokines. These processes accelerate matrix degradation, intensify inflammatory responses in the IVD microenvironment, and induce non-physiological death of NPCs, leading to cell dysfunction and tissue injury (87). Ultimately, the imbalance in oxygen radical metabolism within NPCs disrupts the structural and functional homeostasis of the IVD, causing ECM degradation and water loss, thereby driving the onset and progression of IVDD (88).

Moreover, mitochondria are the primary source of ROS during electron transport chain activity, and excessive ROS levels increase the Bax/Bcl-2 ratio by upregulating the pro-apoptotic protein Bax relative to the anti-apoptotic protein Bcl-2. This altered ratio enhances mitochondrial membrane permeability and compromises its structural integrity, leading to the release of apoptosis-related molecules such as cytochrome c. These molecules subsequently activate the caspase cascade, initiating programmed cell death via the mitochondrial apoptotic pathway (89). Studies have shown that hydrogen peroxide (H_2_O_2_) can induce the expression of microRNA-96-5p, Bax, and cleaved caspase-3, thereby promoting NPCs apoptosis (90). Additionally, oxidative stress stimulates the expression of Early Growth Response 1 (EGR1) in NPCs, which activates the NR4A3 signaling axis, increasing the levels of Bax and caspase-3 while suppressing the expression of Bcl-2, aggrecan, collagen II, and ECM-related proteins, ultimately promoting NPCs apoptosis (91). Oxidative stress also upregulates Beclin-1 expression and facilitates the conversion of LC3B-I to LC3B-II, promoting autophagosome formation and contributing to the degeneration of rat NPCs (92, 93). Animal experiments further reveal that oxidative stress significantly enhances METTL16 expression while downregulating MAT2A in NPCs, disrupting the balance of MAT2A pre-mRNA splicing, maturation, and degradation, thereby accelerating NPCs apoptosis (94). Overall, oxidative stress-induced NPCs apoptosis represents a core mechanism in the development of intervertebral disc degeneration. It is intricately linked to inflammatory pathways and mitochondrial apoptosis, forming a self-amplifying loop that intensifies NPCs death and contributes to the process of LDH reabsorption. The IVD lacks direct blood supply and is in a hypoxic environment, but IVD cells still undergo aerobic metabolism and produce ROS. During IVD degeneration, intracellular metabolites cannot be efficiently transported, leading to the accumulation of metabolic waste, which in turn impairs cell function. Oxidative stress disrupts cellular homeostasis by increasing ROS, triggering the release of pro-inflammatory cytokines and degrading extracellular matrix components. This, in turn, induces apoptosis, autophagy, and senescence in disc cells. Trace elements, as important cofactors in enzyme-catalyzed reactions and redox processes, may contribute to the metabolic imbalance caused by spinal degeneration. Antioxidant trace elements help regulate inflammation and prevent oxidative damage (95), thereby promoting LDH reabsorption.

#### 2.2.8 Mechanisms of cellular autophagy and LDH reabsorption

Autophagy is a coordinated self-degradation mechanism that serves as a protective response against harmful stimuli such as nutrient deprivation and hypoxia (96). It plays a critical role in maintaining cellular homeostasis by removing damaged or excessive ROS, peroxisomes, endoplasmic reticulum, mitochondria, and other cellular components, thereby reducing the accumulation of abnormal proteins and organelles (97). The CEP contributes to delaying degeneration by regulating the PI3K/Akt/autophagy signaling pathway through exosomal communication, which in turn affects the progression of IVDD (98). Moreover, studies by Tu et al. (99) revealed that endoplasmic reticulum stress can promote autophagy and ECM degradation in NPCs, ultimately enhancing NPCs apoptosis. Among autophagy markers, LC3 is widely recognized as a key indicator of autophagic activity and serves as a structural component of the autophagosome membrane (100). The p62 protein binds to LC3 and is selectively incorporated into autophagosomes, where it undergoes effective degradation during the autophagic process (101). Autophagy and apoptosis act on the same set of cellular regulatory proteins, exhibiting a bidirectional effect of both inhibiting and promoting apoptosis. Autophagy in NPCs is closely related to LIVD and reabsorption. When the normal metabolic processes of NPCs are disrupted, it leads to the accumulation of harmful substances and an upregulation of apoptosis levels, thereby accelerating IVDD. Autophagy prevents the occurrence of LIVD by regulating apoptosis and alleviating inflammation, thus mitigating the progression of IVDD (102, 103).

#### 2.2.9 Other factors

**Figure 3:**
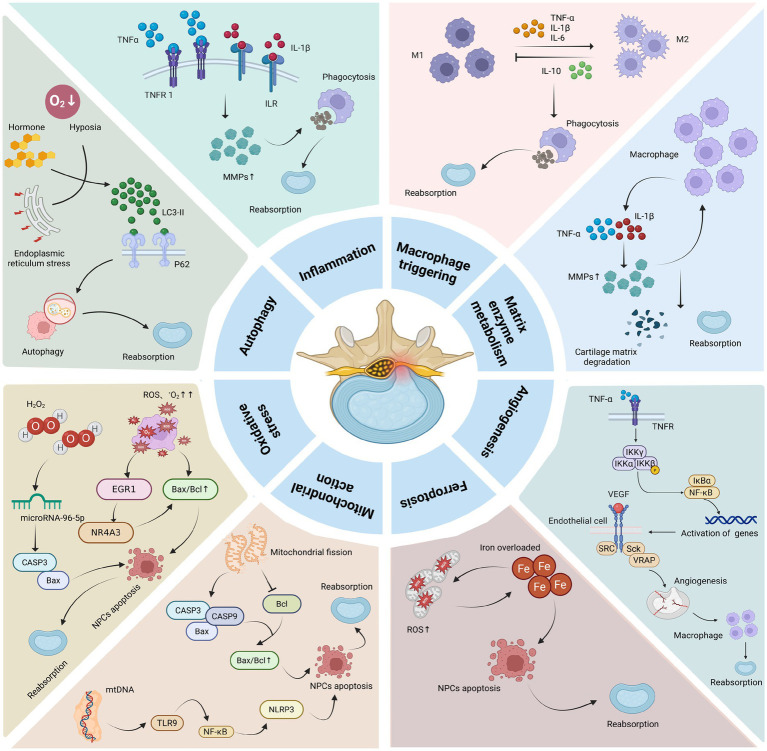
Mechanism of action of LDH reabsorption. Macrophages can secrete pro-inflammatory factors such as TNF-*α*, IL-1*β*, and IL-6, which induce inflammatory responses while also upregulating the expression of MMPs. The latter can, in turn, promote macrophage infiltration. M1 and M2 macrophages can regulate each other through the secretion of different cytokines, ultimately promoting phagocytosis and ECM degradation, thereby facilitating LDH reabsorption. In addition, pro-inflammatory factors can stimulate angiogenesis, which further enhances macrophage infiltration and promotes LDH reabsorption. Oxidative stress and mitochondrial fission can upregulate the expression of Bax/Bcl, promoting NPC apoptosis through NF-κB and other related signaling pathways, and also induce iron overload leading to ferroptosis, which further accelerates NPCs apoptosis and promotes LDH reabsorption. Protective autophagy can regulate NPCs apoptosis and directly promote LDH reabsorption (The graphic was created with BioRender.com).

LDH reabsorption is a complex physicochemical process that not only involves the individual actions of the mechanisms mentioned above but also their interactions. For example, inflammation can stimulate the production of MMPs, which indirectly promotes angiogenesis. The production of MMPs and angiogenesis can further regulate the infiltration of macrophages and other immune cells, thereby modulating the inflammatory response, resulting in a synergistic effect that promotes LDH reabsorption. In addition to the mechanisms mentioned above, resorption may also be influenced by neurotransmitters and mechanical forces. Tu et al. (104) demonstrated that human NPCs, particularly fibrotic NPCs, are innervated by nerve fibers. Speichert et al. (105) found that norepinephrine (NE), under IL-1β stimulation, significantly downregulates the gene expression of Sex Determining Region Y-box 9 (SOX9), type II collagen, cartilage oligomeric matrix protein (COMP), and aggrecan, while upregulating the expression of matrix metalloproteinase 13 (MMP13) and a disintegrin and metalloproteinase with thrombospondin motifs 4 (ADAMTS4) in chondrocytes, thereby promoting apoptosis of NPCs. Studies have shown that calcitonin gene-related peptide (CGRP) can inhibit NPCs proliferation and promote apoptosis, inflammation, and ECM degeneration by activating the NF-κB and MAPK signaling pathways (106). Moreover, vasoactive intestinal peptide (VIP), a sympathetic neurotransmitter, can target fibroblast growth factor 18 (FGF18) to activate the Akt signaling pathway, thereby suppressing apoptosis and inflammatory responses in NPCs (107). Therefore, multiple neurotransmitters play critical roles in the process of LDH reabsorption. Mechanical forces and loading also affect LDH reabsorption. Under low levels of mechanical stress, NPCs exhibit anabolic responses, whereas high levels of stress induce catabolic responses characterized by increased protease expression and activity (108). Thus, high-intensity mechanical stress can promote NPCs apoptosis, thereby accelerating LDH reabsorption. The summary table of LDH reabsorption mechanisms can be found in Table 2. The summary diagram of LDH reabsorption mechanisms can be found in Figure 3.

**Table 2:** Mechanism of action of LDH reabsorption.

Mechanisms	Reference	Study design	Sample	Results
Inflammation response	Xu et al. (42)	Experimental study	Whole blood samples	The inflammatory response can lead to an excess of copper ions triggering cytotoxicity and inducing cell death associated with copper toxicity.
	Djuric et al. (45)	Retrospective observational study	Human IVD	Inflammatory factors such as TNF-α and IL-1β activate the secretion of chemokines by IVD cells, which induces the activation of MMPs and stimulates neointima formation, and the secretion of cytokines to promote LDH reabsorption.
Macrophage triggering	Haro et al. (54)	Experimental study	Rabbit IVD	IVD cells and macrophages secrete cytokines, such as MCP-1, MIP-1α, and TNF-α, which promote macrophage chemotaxis and participate in the release of MMP-3, further promoting reabsorption.
	Ohba et al. (55)	Experimental study	Mouse IVD	TGF-β and TSLP up-regulate MCP-1 expression through the PI3K/Akt pathway, promoting macrophage infiltration and IVD reabsorption.
Metabolic balance of matrix enzymes	Haro et al. (54)	Experimental study	Rabbit IVD	Upregulation of MMP-3 in the MMP family enhances macrophage infiltration, proteoglycan degradation, and IVD reabsorption by promoting the release of macrophage chemotactic factors.
	Haro et al. (60)	Experimental study	Rabbit IVD	Upregulation of MMP expression by TNF-α accelerates proteoglycan loss and cartilage matrix degradation in protruded IVDs, while MMP secretion facilitates the interaction between macrophages and chondrocytes in LDH reabsorption.
Vascularization factors	Minamide et al. (61)	Experimental study	Rabbit IVD	Ruptured IVD tissue promotes macrophage infiltration through neovascularization, thereby, promoting LDH reabsorption.
	Haro et al. (62)	Experimental study	Mouse IVD	
	Kushioka et al. (66)	Review	NA	M1-type macrophages secrete TNF-α and VEGF to promote angiogenesis, and M2-type macrophages secrete MMP9 and PDGF-BB to support stable vessel growth. Neovascularization and macrophage nodes work together to promote LDH reabsorption.
	Lou et al. (67)	Experimental study	Mouse BMDs	
	Spiller et al. (68)	Experimental study	Human macrophage	
Ferroptosis	Dixon et al. (20)	Experimental study	Human cells	Ferroptosis is induced by intracellular iron overload, ROS-dependent accumulation of lipid peroxides
	Zhang et al. (69)	Experimental study	Mouse IVD	Homocysteine-induced oxidative stress can activate ferroptosis and cause substantial loss of NPCs.
	Wang et al. (70)	Experimental study	Human NPCs	Ferroptosis can activate the p38MAPK signaling pathway leading to cell membrane rupture and necrosis, and plays an important role in LIVDD.
	Zhu et al. (71)	Experimental study	Mouse IVD	
Mitochondrion	Hu et al. (80)	Experimental study	Human NP tissues	Excessive mitochondrial fission activates the intrinsic apoptotic pathway, up-regulating the pro-apoptotic proteins caspase-3, caspase-9, and Bax while down-regulating the anti-apoptotic protein Bcl-2, thereby triggering apoptosis of NPCs.
	Lu et al. (81)	Experimental study	Mouse IVD	Mitochondrial DNA activates the TLR9-NF-κB-NLRP3 signaling pathway, which promotes the expression of inflammatory vesicles, leading to cellular pyroptosis and promoting IVDD and LDH reabsorption.
Oxidative stress	Risbud and Shapiro (87)	Review	NA	Oxidative stress causes reactive oxygen species accumulation through an imbalance of oxygen free radicals and antioxidants, leading to the loss of NPCs and IVD substrates.
	Zhang et al. (89)	Experimental study	Mouse kidney	Excess ROS cause apoptosis of NPCs by up-regulating the Bax/Bcl-2 ratio, activating the mitochondrial apoptotic pathway, releasing pro-apoptotic molecules, such as cytochrome C, and activating the caspase cascade reaction.
	Wang et al. (90)	Experimental study	Human NPCs	H_2_O_2_ induces the microRNA-96-5p, apoptotic protein Bax and cleaved-caspase3 expression, thereby promoting apoptosis of NPCs.
	Zheng et al. (91)	Experimental study	Human NP tissue	In addition, oxidative stress further promotes NPCs apoptosis through the EGR1-NR4A3 signaling axis, pro-autophagy, and ultimately promotes LDH reabsorption.
Autophagy	Mizushima et al. (97)	Review	NA	Cellular autophagy is a protective cellular response to harmful stimuli, maintaining homeostasis by removing damaged organelles and oxygen free radicals.
	Luo et al. (98)	Experimental study	Mouse NPCs	Autophagy is regulated through the PI3K/AKT pathway and promotes apoptosis in NPCs.
	Tu et al. (99)	Experimental study	Human NPCs	Endoplasmic reticulum stress activates autophagy and promotes apoptosis of NPCs.
	Vujic et al. (100)	Experimental study	Mouse macrophage	The detection of autophagy markers LC3 and P62 suggests that cellular autophagy is closely related to LIVD disease and reabsorption.
Others	Speichert et al. (105)	Experimental study	Human articular cartilage	NE promotes apoptosis of NPCs by down-regulating SOX9, type II collagen and up-regulating MMP13 and ADAMTS4 expression.
	Sun et al. (106)	Experimental study	Mouse	CGRP inhibits cell proliferation and promotes apoptosis through activation of NF-κB and MAPK signaling pathways.
	Sun et al. (107)	Experimental study	Mouse LIVD	VIP inhibits apoptosis and inflammatory response of NPCs through Akt signaling pathway.
	Fearing et al. (108)	Retrospective study	NA	Mechanical forces and loading drive LDH reabsorption by enhancing protease expression, activity, and promoting apoptosis in NPCs.

### 2.3 Research progress on LDH treatment

#### 2.3.1 Mechanisms of action of basic therapy in LDH reabsorption

LDH resorption refers to the spontaneous shrinkage or disappearance of protruded NP tissue in patients with LDH without undergoing surgery, chemonucleolysis, or endoscopic NP removal. Conventional conservative therapies in clinical practice include health education, dietary regulation, exercise therapy, and rehabilitation treatment. LDH is a chronic condition, and the resulting persistent pain can induce anxiety by altering brain function and structure (109). Pain and anxiety share common neural substrates, contributing to their close association (110). Pain is a multidimensional experience that involves sensory-discriminative, affective-motivational, and cognitive-evaluative components (111). Pain is commonly present throughout the course of LDH, often impairing mental health and diminishing quality of life. It can lead to dysfunctional symptoms such as depression, anxiety, pain catastrophizing, and rumination. Preoperative interventions targeting patients’ catastrophic thoughts can help them cope more effectively with pain and accelerate recovery (112). Furthermore, postoperative education via mobile applications significantly enhances patients’ understanding of LDH, leading to improved attitudes and practical self-management skills (113). Therefore, providing psychological counseling and education to LDH patients is beneficial at all stages of clinical diagnosis and treatment.

IVD is the largest avascular tissue in the human body, and its nutrient supply primarily relies on diffusion from peripheral capillaries and the endplates. The notochordal phenotype of NP cells disappears during adolescence, accompanied by cell death and chondrocyte proliferation. As the largest avascular organ in the body, the IVD is particularly susceptible to nutrient deficiency, which may contribute to disc degeneration. During aging and degenerative processes, IVD cells undergo senescence and growth arrest, leading to the release of pro-inflammatory cytokines and matrix-degrading enzymes (114), ultimately resulting in IVDD. Relevant studies have shown that adequate carbohydrate intake during mountain marathons can limit markers such as creatine kinase, lactate dehydrogenase, and aspartate aminotransferase, thereby reducing exercise-induced muscle damage and physiological load (115). Properly managed exercise training has been shown to relieve pain in non-surgically treated patients and partially restore motor function (116). For LDH patients suffering from neuropathic pain, yoga-based interventions aimed at enhancing mobility, core strength, and spinal and hamstring flexibility can significantly reduce pain and disability (117). Furthermore, rehabilitation studies following unilateral microdiscectomy have revealed that long-term regular physical activity helps alleviate pain, accelerates postoperative recovery, and plays a vital role in improving quality of life and preventing occupational impairment (118). In summary, conservative clinical treatments for LDH-emphasizing mental well-being, exercise, and rehabilitation-may promote LDH resorption by limiting muscle injury biomarkers such as creatine kinase and lactate dehydrogenase, thus achieving improved therapeutic outcomes.

#### 2.3.2 Mechanism of action of orally administered drugs in LDH reabsorption

In clinical practice, conservative treatment of LDH primarily relies on oral pharmacotherapy. Commonly prescribed medications include non-steroidal anti-inflammatory drugs (NSAIDs), opioids, muscle relaxants, analgesics, glucocorticoids, and neurotrophic agents, which mainly function to relieve pain and suppress inflammation. Resorption is frequently observed in cases of large IDH, where enzymatic degradation and phagocytosis of cartilaginous tissues are driven by IVDD, inflammatory responses, and neovascularization within the herniated tissue. The proliferation of cells and blood vessels within the herniated LIVD is a key factor promoting the resorption process (119). NSAIDs are commonly recommended for short-term use in patients with LBP without nerve root compression (120), demonstrating good efficacy in relieving pain and suppressing postoperative inflammatory pain. Compared to opioids, NSAIDs are more economical and have fewer side effects. Their therapeutic effect in LDH is mediated by inhibition of COX, which reduces prostaglandin E2 synthesis in neural cells and thereby alleviates inflammation-related pain. However, NSAIDs may pose risks to gastrointestinal and renal health, particularly in elderly patients (121). As such, in patients with a history or risk of peptic ulcers, co-administration of COX-2 inhibitors or enteric-coated aspirin is recommended to minimize gastrointestinal side effects (122, 123). Diclofenac sodium, a widely used NSAID, also exerts its effects by inhibiting COX-2 activity, thereby reducing local pain and restoring lumbar spine mobility (124).

For lumbosacral pain that is poorly controlled by NSAIDs, opioid analgesics and muscle relaxants may be considered. Opioids are effective in relieving moderate to severe pain and can moderately improve motor function. However, they are associated with numerous side effects, including respiratory depression, potential drug dependence, nausea, dizziness, headache, and drowsiness. As such, they are typically reserved for short-term treatment and should be used with caution (125). When patients exhibit significant pathological changes or experience severe pain that is unresponsive to other medications, muscle relaxants are often used as alternatives. These agents relieve muscle spasms, help restore range of motion, and improve pain tolerance, while having fewer side effects and being suitable for short-term use. Glucocorticoids alleviate nerve root compression and inflammation caused by herniated IVDs by suppressing the activation of inflammatory cells and the release of inflammatory mediators. Inflammatory cytokines such as IL-6 and TNF-*α* play pivotal roles in the pathogenesis of LDH, and glucocorticoids help reduce their levels (126). Additionally, glucocorticoids can regulate immune responses, and their long-term use may promote neovascularization by persistently inhibiting inflammatory and immune processes, thereby indirectly facilitating the resorption of the herniated NP (127). Most patients with massive LDH prefer non-surgical treatment. The resorption of IDH is closely related to capillary infiltration of the large, free herniated tissue. VEGF, a major pro-angiogenic factor, promotes neovascularization at the edges of the IVD and facilitates disc resorption. Clinically, a combination of oral celecoxib, spinal cord demyelination decoction, and acupuncture is used. This integrated approach-combining oral and external therapies-enhances NP resorption and reduces nerve root edema. More than half of patients treated with this regimen exhibit evidence of LIVD resorption (128).

Recent studies have revealed that the deficiency of brain-derived neurotrophic factor (BDNF) can cause neurodegeneration in central motor structures, subsequently triggering motor neuron diseases (129). Nerve growth factor, initially investigated for its capacity to promote neuronal growth and treat neurological disorders (130), has been shown to repair damaged neural tissues. Among these neurotrophic factors, BDNF plays a crucial role in the development of the central nervous system by promoting neuronal survival, growth, differentiation, and regeneration, as well as facilitating remyelination of injured neurons (131). Damage to LIVD and endplates can result in pathological nerve ingrowth. Herniation of IVDs compressing nerve roots may lead to traumatic neuropathic pain. Such injuries activate lumbar sensory receptors, initiating inflammation, upregulating IL-1β and TNF-*α* in degenerative disc tissue, and enhancing neovascularization (132, 133), thereby alleviating pain and accelerating disc resorption through improved blood supply. Additionally, Zhou et al. (134) found in a rabbit IVD model that midkine (MK) treatment led to a significantly greater reduction in disc weight, increased neovascularization, and elevated inflammatory cell infiltration compared to controls. MK also accelerated IVD degradation and promoted resorption. As a heparin-binding growth and differentiation factor, MK plays a key role in angiogenesis and wound healing. Epidural MK injection has shown therapeutic promise for LDH, outperforming conventional treatments such as NSAIDs and intrathecal corticosteroids (135, 136). With advancements in technology and pharmacological delivery, novel minimally invasive treatments-such as radiofrequency ozone therapy and targeted injections of anti-inflammatory agents into the nerve root foramina-are emerging as safe and effective options (137). Although pharmacotherapy effectively reduces inflammation and pain, anti-inflammatory drugs like NSAIDs and glucocorticoids, whether administered systemically or locally, may impair the body’s natural healing process, prolonging the duration of LDH and causing adverse effects on renal, gastrointestinal, and cardiovascular systems (138), which require further research to address.

#### 2.3.3 Mechanism of action of biological agents in LDH reabsorption

##### 2.3.3.1 Platelet rich plasma

Treatment with biological agents can significantly improve pain symptoms, physical function, and overall quality of life in patients with IVD disorders. Commonly used biological agents in clinical practice include mesenchymal stem cells (MSCs), platelet-rich plasma (PRP), and α2-macroglobulin, all of which exhibit significant potential in promoting chondrogenesis within the IVD. These agents are highly effective in treating IVD-related LBP and lower limb pain, with minimal associated risk, resulting in notable improvements in patients’ pain relief, physical capabilities, and quality of life (139, 140). PRP therapy utilizes platelet concentrates obtained from autologous whole blood via centrifugation. Upon activation, the platelets release large quantities of growth factors, such as transforming growth factor, VEGF and platelet-derived growth factor (141, 142). These concentrated platelets are directly injected into the IVD, initiating a cascade of healing responses that promote tissue repair and regeneration, reduce inflammation, and aid in the repair of damaged nerves (143). Clinical studies have shown that IVD herniation is a leading cause of LBP. PRP has been demonstrated to significantly alleviate LBP in patients with IVD herniation, with long-lasting therapeutic effects, representing a safe and promising alternative to epidural local anesthetics and corticosteroids (144, 145).

In IVDD, there is a reduction in the synthesis of proteoglycans and type II collagen, alongside an increase in type I collagen synthesis. Herniation of the NP, which compresses the nerve roots, is frequently accompanied by inflammatory responses. The major inflammatory mediators originate from degenerative disc cells and include cytokines such as p38 MAPK, COX-2, and TNF-*α*. TNF-α is also secreted by endoneurial macrophages and neuroglial cells. Once activated, TNF-α stimulates the dorsal root ganglion to produce a large number of inflammatory mediators, resulting in neuropathic pain. Additionally, when TNF-α and IL-1β act on degenerative disc cells, they increase apoptosis rates and promote neovascularization and nerve ingrowth into the degenerated disc, exacerbating pain (146–148). PRP, through the anti-inflammatory and immunomodulatory actions of its platelets, regulates growth and immune factors in damaged nerves, thereby reducing inflammation and pain and facilitating the resorption of LDH.

##### 2.3.3.2 Mesenchymal stem cell

MSCs, first discovered in the 20th century and derived from periosteum, adipose tissue, and muscle, possess multidirectional differentiation potential, immunomodulatory capacity, and regenerative properties. These characteristics enable them to promote osteogenesis, chondrogenesis, and tissue repair, making them widely used in muscle recovery, meniscus tears, tendon and ligament injuries, and IVDD, where they have shown potential for cartilage regeneration and alleviating osteoarthritis. MSCs regulate immune responses and promote tissue repair by secreting growth factors and nutrients such as VEGF. In addition, they exert immunomodulatory and anti-inflammatory effects by inhibiting dendritic cell maturation, T and B lymphocyte activation, and reducing the cytotoxicity of natural killer cells (149, 150). MSCs are undifferentiated pluripotent cells capable of self-renewal and differentiation, and have been widely applied in various medical fields (151). Studies have shown that regenerative stem cell therapies are particularly suitable for treating musculoskeletal disorders, as they promote bone growth and fusion as well as cartilage regeneration. These therapies also facilitate IVD cartilage regeneration and have demonstrated significant therapeutic effects in lumbar spine disorders. MSCs can be isolated from bone marrow, synovium, adipose tissue, umbilical cord, and other tissues. With appropriate intervention, they can differentiate into bone, cartilage, fat, and other mesenchymal tissues (152), thereby contributing to the treatment of LDH by promoting reabsorption. In a clinical study on MSC therapy for IVDD, direct injection of bone marrow-derived MSCs (BM-MSCs) into the NP led to significant relief of LBP, improved quality of life, and increased water content in the lumbar intervertebral disc as shown by MRI (153). In studies using autologous biological agents to repair damaged tissues, BM-MSCs have been applied for their anti-inflammatory, immunomodulatory, and regenerative properties. Their therapeutic efficacy and safety have been investigated in patients with severe chronic LBP. Interventions targeting multiple structures, including IVD, facet joints, nerve roots, and sacroiliac joints, based on patients’ clinical symptoms, showed favorable outcomes after treatment (154). In summary, MSCs are undifferentiated pluripotent cells that can promote symptom relief and disc reabsorption in patients with LDH through their self-renewal and differentiation capabilities.

#### 2.3.4 Other therapies

**Figure 4:**
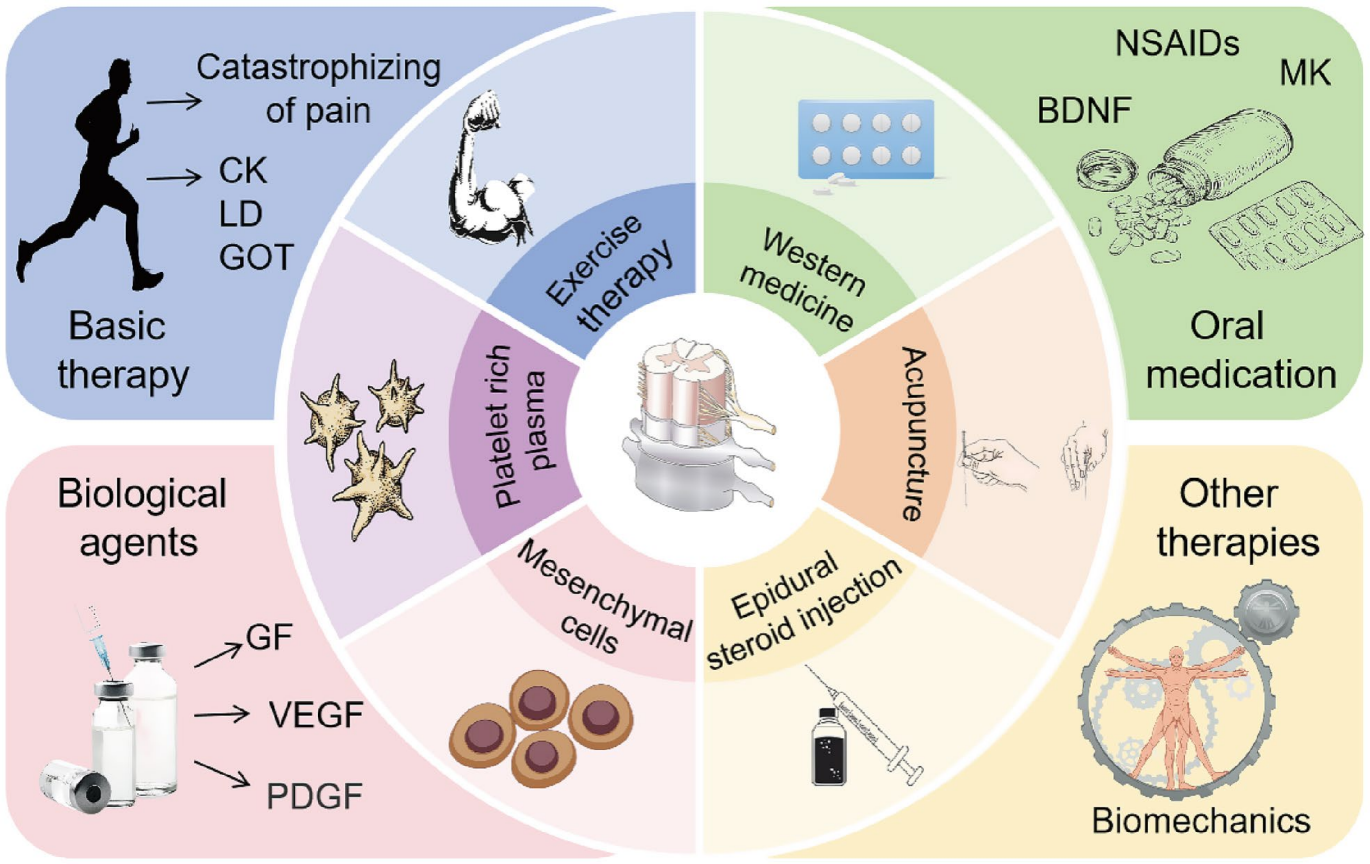
Common conservative clinical treatments to promote LDH reabsorption. Rational exercise control training can alleviate pain in patients, reduce muscle damage and exercise load, thereby promoting LDH reabsorption. Oral medications such as NSAIDs, corticosteroids, or opioids, as well as Western medical treatments like MK epidural injections, exert anti-inflammatory effects, relieve pain, and promote LDH reabsorption. The application of biological agents such as (PRP) and MSCs can exhibit anti-inflammatory properties, regulate immune responses, promote tissue repair, and enhance LDH reabsorption. Epidural steroid injections, lumbar traction, massage therapy, and acupuncture can relieve lower back pain, improve lumbar function, and further promote LDH reabsorption.

LDH has a complex etiology and involves multiple pathogenic mechanisms, with a variety of clinical treatment options available. Among these, epidural steroid injections are widely used to treat LDH due to their significant anti-inflammatory effects, pain relief, and improvement in lumbar function. They are particularly effective in alleviating radicular pain and axial LBP associated with LDH (155, 156). In clinical studies, transforaminal steroid injections into the lumbar spine effectively relieved radicular pain, with more than half of the patients experiencing pain reduction, functional improvement, and high satisfaction rates (157). Hong et al. (158) conducted MRI evaluations before and after treatment in LDH patients who received transforaminal epidural steroid injections. The results showed that most patients experienced a reduction in herniated IVD volume, and even in cases without significant MRI changes, clinical symptoms improved, thereby avoiding the trauma and cost associated with surgery. Furthermore, changes in the human lumbar spine are neither singular nor isolated. The onset of LDH is closely associated with structural abnormalities such as facet joint hypertrophy, loss of IVD height, and the formation of adjacent osteophytes. There are complex biomechanical interactions among the lumbar spine, adjacent joints, and surrounding muscles. Massage therapy guided by musculoskeletal assessment models has been shown to effectively relieve pain and reduce functional impairment in LDH patients (159). Lumbar traction, which improves spinal biomechanics, can relieve pain and enhance lumbar mobility in LDH patients. It also significantly reduces serum concentrations of inflammatory cytokines such as IL-6 and IL-8 following treatment (160). Mechanical traction has demonstrated significant efficacy in alleviating lumbar and leg pain, improving clinical symptoms, and reducing the need for surgical intervention in LDH patients (161). In addition, conservative treatments such as acupuncture and moxibustion also show therapeutic benefits in LDH, primarily by modulating the biomechanical relationships between the lumbar spine and adjacent tissues (162, 163). The summary table of research progress on conservative treatment for LDH reabsorption can be found in Table 3. The summary diagram of research progress on conservative treatment for LDH reabsorption can be found in Figure 4.

**Table 3:** Research progress in LDH treatment.

Therapy	References	Study design	Number of patients	Mechanisms
Basic therapy	Gatchel et al.(109)	Review	NA	Persistent pain of LDH can induce anxiety by altering brain function and structure
	Ionescu et al. (112)	Research	190	Preoperative interventions targeting catastrophic thoughts can improve pain coping and promote recovery.
	Yurube et al. (114)	Review	NA	Aging and degeneration cause IVD cell senescence, releasing pro-inflammatory cytokines and enzymes, leading to IVDD
	Viribay et al. (115)	Randomized controlled trial	20	Adequate carbohydrate intake during mountain marathons helps reduce exercise-induced muscle damage and physiological load by limiting markers like creatine kinase, lactate and dehydrogenase, promoting LDH reabsorption
Oral administration of Western medicine	Peck et al. (121)	Review	NA	NSAIDs alleviates inflammation-related pain through reducing prostaglandin E2 synthesis in neural cells by inhibition of COX
	Price et al. (125)	Review	NA	Opioids effectively relieve moderate to severe pain and improve motor function but have side effects like respiratory depression, dependence, and nausea, making them suitable for short-term use.
	Martinez et al. (126)	Review	NA	Glucocorticoids reduce nerve root compression and inflammation in LDH by suppressing inflammatory cells and cytokines like IL-6 and TNF-α
	Chen et al. (131)	Experimental study	NA	BDNF promotes neuronal survival, growth, differentiation, regeneration, and remyelination, playing a key role in central nervous system development.
	Xiong et al. (132)	Review	NA	IVD herniation compresses nerve roots, causing neuropathic pain, inflammation, and increased IL-1β, TNF-α,as well as neovascularization in degenerative disc tissue.
	Yoshida et al.(135)	Experimental study	NA	MK accelerates the degradation rate of IVD and promotes reabsorption.
Platelet rich plasma	Everts et al. (141)	Review	NA	Activated platelets release growth factors like TGF, VEGF, and PDGF, which promote tissue repair and regeneration.
	Tuakli-Wosornu et al. (143)	Randomized controlled trial	47	Concentrated platelets injected into the IVD promote tissue repair, reduce inflammation, and aid nerve regeneration through a healing response cascade, ultimately promoting LDH reabsorption.
	Wang et al. (146)	Observational study	NA	In IVDD, reduced proteoglycan and type II collagen synthesis, along with increased type I collagen, triggers inflammation via cytokines like TNF-α, leading to neuropathic pain, apoptosis, and nerve ingrowth.
Mesenchymal stem cell	Trapana et al. (149)	Review	NA	MSCs, derived from tissues like periosteum and adipose, possess regenerative, immunomodulatory, and differentiation capabilities. They promote tissue repair, osteogenesis, and chondrogenesis, as well as alleviate conditions like osteoarthritis, by secreting growth factors and modulating immune responses.
	Vadala et al. (150)	Review	NA	
	Piccirilli et al. (151)	Randomized controlled trial	22	MSCs are undifferentiated pluripotent cells capable of self-renewal and differentiation, having been widely applied in various medical fields
	Oehme et al. (152)	Review	NA	Regenerative stem cell therapies are effective for musculoskeletal disorders, promoting bone growth, fusion, and cartilage regeneration. MSCs, isolated from tissues like bone marrow and adipose, can differentiate into various mesenchymal tissues, aiding in IVD and lumbar spine regeneration.
Other therapies	Rivera CE et al. 2018 (155)	Review	NA	Epidural steroid injections effectively treat LDH by reducing inflammation, relieving pain, and improving lumbar function, especially for radicular pain.
	Smith et al. (157)	Review	NA	Transforaminal steroid injections into the lumbar spine effectively reduce radicular pain, improving function and achieving high patient satisfaction.
	Cao et al (160)	Randomized controlled trial	66	Lumbar traction relieves pain, enhances lumbar mobility, and reduces inflammatory cytokines like IL-6 and IL-8 in LDH patients.
	Li et al (162)	Meta-analysis	NA	Conservative treatments like acupuncture and moxibustion benefit LDH by modulating biomechanical relationships between the lumbar spine and surrounding tissues.

## 3 Conclusion

LDH reabsorption exists at various stages of the onset and progression of LDH, being more pronounced in patients with large and free types of LDH. Relevant studies have shown that conservative treatments, including health education, dietary regulation, exercise therapy, oral and topical medications, and biologics such as epidural steroid injections, can modulate mechanisms such as ferroptosis, mitochondrial fission, oxidative stress, tissue vascularization, inflammatory response, macrophage activation, apoptosis, and autophagy of NPCs, thereby alleviating LDH pain, improving lumbar function, and promoting LDH reabsorption to treat LDH effectively. Early implementation of conservative treatment can promote LDH reabsorption, thus avoiding the heavy burden that surgical treatments may place on families and society. Therefore, systematic research into the mechanisms of LDH reabsorption and the implementation of relevant conservative treatments are of significant importance and value for the treatment of LDH.
